# Advancements in Regenerative Therapies for Orthopedics: A Comprehensive Review of Platelet-Rich Plasma, Mesenchymal Stem Cells, Peptide Therapies, and Biomimetic Applications

**DOI:** 10.3390/jcm14062061

**Published:** 2025-03-18

**Authors:** Andrew J. Goulian, Brielle Goldstein, Maarouf A. Saad

**Affiliations:** 1 College of Medicine, California Northstate University, Elk Grove, CA 95757, USA; andrew.goulian9114@cnsu.edu (A.J.G.); brielle.goldstein9828@cnsu.edu (B.G.); 2 Department of Orthopaedic Surgery, University of California, Sacramento, CA 95817, USA

**Keywords:** regenerative medicine, orthopedics, platelet-rich plasma, PRP, mesenchymal stem cells, biomimetics, peptide therapy, tissue healing

## Abstract

**Background/Objectives**: Regenerative therapies have gained interest in orthopedic applications for their potential to enhance tissue regeneration, functional recovery, and pain modification. This review evaluates the clinical efficacy of platelet-rich plasma (PRP), mesenchymal stem cells (MSCs), peptide-based treatments, and biomimetic materials in orthopedic care, with a focus on pain reduction and functional outcomes. **Methods**: A structured literature search in PubMed (January 2009–January 2025) identified 160 studies. After applying inclusion criteria prioritizing randomized controlled trials (RCTs) and clinical trials, 59 studies were included: 20 on PRP, 20 on MSCs, 10 on peptide therapies, and 7 on biomimetics. Data extraction focused on pain reduction and functional recovery, with risk of bias assessed using the Cochrane Risk of Bias (RoB) tool and ROBINS-I tool. A random-effects meta-regression analysis was conducted to evaluate the impact of therapy type, sample size, and risk of bias on reported pain reduction outcomes. **Results**: Meta-regression analysis identified MSC therapy as the most effective intervention for pain reduction (β = 8.45, *p* < 0.05), with PRP and peptide-based therapies showing moderate improvements, and biomimetic therapies demonstrating the lowest effect. PRP provided short-term pain relief, particularly in acute injuries and tendon repair, though inconsistencies in preparation methods limited success in chronic conditions. MSC therapies demonstrated cartilage regeneration and early osteoarthritis improvement, but high costs and ethical concerns remain barriers to widespread adoption. Peptide-based therapies and biomimetic materials, including engineered scaffolds and autologous protein solutions, showed promise for infection control and wound healing, though further research is needed to optimize dosing, delivery methods, and long-term safety. **Conclusions**: Regenerative therapies offer significant potential in orthopedic care, with MSC therapies demonstrating the most reliable regenerative effects, PRP providing short-term symptomatic relief, and peptide-based and biomimetic treatments emerging as promising adjuncts. However, standardized protocols and large-scale clinical trials are needed to establish long-term efficacy and improve clinical translation for broader adoption.

## 1. Introduction

Orthopedic surgery often involves managing complex musculoskeletal injuries and degenerative conditions that pose challenges in achieving complete functional recovery. Traditionally, treatments such as surgical repair, physical therapy, and pharmaceutical interventions have been employed to address these conditions, but they frequently result in variable outcomes and inconsistent recovery periods [1]. As a result, regenerative therapies have gained considerable attention in recent years for their potential to accelerate healing, restore function, and, in some cases, provide alternative solutions to surgery [2]. Among these therapies, platelet-rich plasma (PRP), mesenchymal stem cell (MSC) treatments, peptide-based interventions, and biomimetic applications were selected for this review due to their widespread clinical application, ongoing research, and potential to address distinct aspects of orthopedic regeneration [3,4,5]. PRP and MSC therapies are among the most studied regenerative treatments for musculoskeletal injuries, while peptide-based interventions and biomimetic materials represent newer, promising strategies aimed at enhancing infection control, tissue integration, and functional recovery [2,6].

**Figure 1:**
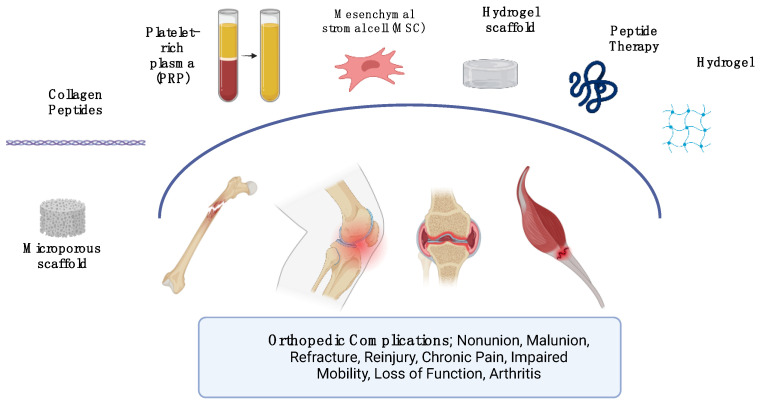
Visual overview of regenerative therapies in orthopedics. Created with BioRender.com (accessed 27 January 2025).

This comprehensive review focuses on PRP, MSCs, peptide-based therapies, and biomimetic materials, as these four approaches represent the most clinically relevant and actively researched regenerative strategies in orthopedic medicine. PRP and MSC-based therapies are among the most widely studied, with extensive clinical applications in musculoskeletal repair. However, despite their widespread use, both therapies face limitations regarding standardization, efficacy variability, and cost. Peptide-based interventions and biomimetic materials, while emerging, introduce novel regenerative strategies that complement PRP and MSC applications by enhancing infection control, promoting wound healing, and improving tissue integration [7]. As illustrated in Figure 1, these regenerative approaches form an interconnected framework, highlighting their distinct roles while demonstrating their potential synergistic applications in orthopedic practice.

Despite the increasing number of studies on regenerative medicine in orthopedics, the field remains fragmented, with inconsistent findings across trials and a lack of standardization in therapy preparation and clinical application [2]. Existing reviews have largely focused on individual modalities rather than integrating and analyzing findings across multiple approaches. This review seeks to bridge this gap by synthesizing results from clinical trials and observational studies across four regenerative strategies, providing a comparative evaluation to determine their effectiveness in different orthopedic applications [8].

PRP therapy, which uses autologous platelets concentrated to deliver growth factors at injury sites, has been extensively studied for its potential to aid in soft tissue and bone repair [9,10,11,12]. However, PRP’s therapeutic efficacy varies, largely due to inconsistent preparation methods and differing platelet concentrations, which can significantly influence treatment outcomes [13,14]. In tendon injuries and partial-thickness rotator cuff tears, PRP has shown potential for short-term improvement, yet the effects often diminish over time, highlighting the need for further refinement in preparation techniques to extend its benefits [15,16].

Similarly, MSC-based therapies, particularly those utilizing MSCs derived from bone marrow, adipose tissue, or umbilical cord blood, offer substantial regenerative potential due to their ability to differentiate into various tissue types and modulate immune responses [17,18]. Clinical applications of MSCs in cartilage repair, early-stage osteoarthritis, and fracture healing have shown encouraging results, especially in early degenerative conditions [19,20,21]. The immunomodulatory properties of MSCs make them adaptable across diverse injury types, yet challenges persist regarding their accessibility and ethical implications, which restrict MSC therapies from achieving broader clinical adoption [22].

Peptide-based therapies, which encompass antimicrobial peptides and growth factors, are particularly appealing for their dual ability to combat infection and promote healing [23,24]. These therapies are increasingly integrated into biomaterials for coating orthopedic implants or utilized as adjunctive treatments for enhanced infection control and tissue regeneration [25,26]. However, optimal dosage and delivery methods for peptides remain under-researched, indicating the need for further study to fully determine their role in orthopedic interventions [27].

Biomimetic materials, including engineered scaffolds and autologous protein solutions, represent an emerging class of regenerative therapies aimed at mimicking natural tissue environments to facilitate healing and integration [28,29]. Engineered scaffolds are especially valuable in joint repair, as they offer structural support while promoting cellular proliferation and matrix deposition [30,31]. Meanwhile, autologous protein solutions, derived from the patient’s own blood, concentrate regenerative proteins and cytokines, providing an option for sustained pain relief and functional improvement [32]. Despite promising results, biomimetics are still in the experimental stages, and more extensive clinical trials are necessary to verify their long-term effectiveness and safety [33].

This review provides a critical synthesis of the strengths, limitations, and clinical applications of these four regenerative strategies, addressing the ongoing challenges of standardization, treatment efficacy, and long-term viability. By offering a comparative assessment, this review aims to inform future research directions and guide clinicians in optimizing regenerative medicine approaches for orthopedic care.

While previous reviews have explored individual regenerative therapies in orthopedics, few provide a comparative analysis across multiple regenerative strategies [2,6]. This review distinguishes itself by synthesizing clinical evidence from PRP, MSC-based therapies, peptide-based interventions, and biomimetic materials to offer a cross-comparative evaluation of their efficacy, mechanisms, and limitations. By critically assessing the standardization challenges and clinical inconsistencies across these therapies, this review highlights key gaps in research and clinical translation. Additionally, this work underscores the ongoing need for treatment standardization, particularly in PRP preparation and MSC sourcing, which remain inconsistent across studies. By providing an integrated analysis of these four regenerative approaches, this review aims to inform clinicians on how to optimize regenerative medicine applications for musculoskeletal repair.

## 2. Methods

### 2.1. Clinical Study Selection

This comprehensive review evaluated the clinical application of regenerative therapies, including PRP, mesenchymal stem cell treatments, peptide-based therapies, and biomimetic materials, in orthopedic medicine. A structured literature search was conducted in PubMed from January 2009 to January 2025, adhering to Preferred Reporting Items for Systematic Reviews and Meta-Analyses (PRISMA) methodology to ensure transparency in study selection [34]. The search strategy incorporated Medical Subject Headings (MeSH) terms and keywords, including “platelet-rich plasma”, “PRP”, “stem cell”, “mesenchymal stem cells”, “peptide therapy”, “biomimetic materials”, and “orthopedic procedures”. The search was restricted to clinical trials and randomized controlled trials (RCTs). Following the initial identification of relevant studies, duplicates were removed, and titles and abstracts were screened to exclude ineligible articles.

**Figure 2:**
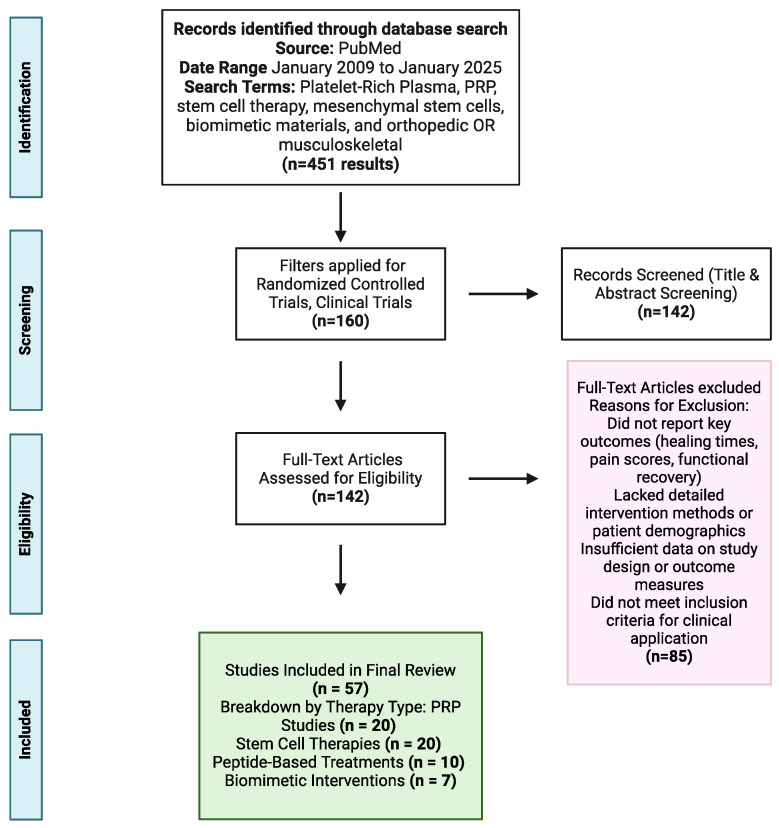
PRISMA flow diagram of study selection process. Created with BioRender.com (accessed on 27 January 2025).

As outlined in Figure 2, the study selection process followed PRISMA guidelines, systematically screening studies for relevance to clinical orthopedic applications of PRP, MSCs, peptide-based therapies, or biomimetic materials. Inclusion criteria required studies to report key clinical outcomes, such as tissue healing times, infection rates, functional recovery, and quality-of-life measures. Preference was given to studies providing detailed descriptions of therapy preparation, intervention protocols, and patient outcomes. Studies were excluded if they were preclinical such as animal-based or in vitro, lacked relevant orthopedic outcome data, or were not published in English.

### 2.2. Risk of Bias Assessment

The risk of bias assessment was conducted using the Cochrane Risk of Bias tool for randomized controlled trials and the Risk of Bias in Non-randomized Studies of Interventions (ROBINS-I) tool for non-randomized and observational studies [35,36]. Each study was evaluated for potential bias across six key domains: selection bias, assessed through random sequence generation and allocation concealment; performance bias, determined by blinding of participants and personnel; detection bias, based on blinding of outcome assessment; attrition bias, which considered incomplete outcome data; and reporting bias, which examined selective reporting.

Each domain was categorized as low risk, moderate risk, serious risk, or critical risk of bias, following the respective tool guidelines, and used collectively for comparative review. Studies with critical risk in any domain were classified as having an overall critical risk of bias and were deemed unreliable for synthesis. Those with at least one serious risk domain were categorized as serious risk, while studies rated as moderate risk in multiple domains but without serious or critical risk were assigned a moderate overall rating. Studies with low risk across all domains were classified as low risk. A detailed summary of these assessments is provided in Supplementary Materials.

### 2.3. Clinical Study Data

Data extraction focused on identifying clinical outcomes such as infection rates, tissue healing, functional recovery metrics, and safety profiles. Studies were screened and categorized based on therapy type: PRP, stem cell therapies, peptide-based treatments, and biomimetic materials. PRP studies were examined for formulation differences, such as leukocyte-rich vs. leukocyte-poor, administration strategies including single vs. multiple injections, and with or without adjunctive treatments. Stem cell therapies were classified according to cell source including bone marrow-derived, adipose-derived, and umbilical cord-derived, and delivery method, such as intra-articular injection, scaffold-assisted implantation, and systemic administration. Peptide-based treatments were evaluated for peptide type, dosage, and application sites, while biomimetic materials were analyzed for composition, structural characteristics, and integration with native tissue. A structured data extraction approach was employed, capturing study design, sample size, intervention details, and outcome measures.

Quantitative synthesis focused on pain reduction and risk of bias across the four therapy types, as pain reduction was the most consistently reported outcome across studies. Detailed data extraction tables were compiled to facilitate structured comparisons, outlined in Supplementary Materials.

### 2.4. Statistical Analysis

To evaluate differences in pain reduction across therapy types while accounting for between-study heterogeneity, a random-effects meta-regression analysis was conducted using the restricted maximum likelihood (REML) method [37,38]. The model assessed the impact of therapy type (PRP, stem cells, peptides, biomimetic), study sample size, and risk of bias on reported pain reduction outcomes. Therapy type and risk of bias (categorized as Low, Moderate, and Serious) were included as categorical moderators, while sample size was treated as a continuous covariate to determine its influence on the variability of reported outcomes.

To evaluate overall differences in pain reduction across therapy types, a one-way analysis of variance (ANOVA) was performed. Normality was assessed using the Shapiro–Wilk test (W = 0.99, *p* > 0.05), confirming normally distributed residuals, while variance homogeneity was tested using Levene’s test (F(3, 43) = 3.64, *p* < 0.05), indicating significant heterogeneity. Due to this violation, Welch’s ANOVA was employed as a robust alternative. Games–Howell post hoc tests were conducted for pairwise comparisons, as they do not assume equal variances.

For meta-regression, biomimetic therapy was selected as the reference category, as it demonstrated the lowest mean pain reduction and provided a baseline for comparison across other therapy types. The Omnibus Test of Moderators was performed to evaluate whether therapy type, sample size, and risk of bias significantly contributed to differences in reported pain reduction. Heterogeneity was assessed using I^2^, with values exceeding 75% indicating substantial unexplained variability [39].

In addition, Fisher’s Exact Test was used to assess whether the distribution of risk of bias significantly differed across therapy types. The final dataset included 20 PRP studies, 20 stem cell studies, 10 peptide studies, and 7 biomimetic studies. Pain reduction was the most consistently reported outcome across studies, with 10 studies omitted due to missing or unreported values. As these omissions were driven by study-specific reporting practices rather than systematic bias, their impact on the overall findings was considered minimal. All statistical analyses were performed in R (version 4.4.2) and statistical significance was set at *p* < 0.05 for all tests.

## 3. Results

This clinical review evaluates the role of regenerative therapies, including platelet-rich plasma, mesenchymal stem cells, peptide-based treatments, and biomimetic applications, in orthopedic surgery. The heterogeneity in study designs, patient populations, and treatment protocols reflects a major challenge in interpreting findings across trials. Table 1 provides a comprehensive summary of the included studies, consolidating key study characteristics, intervention methods, clinical outcomes, and risk of bias assessments. For a more detailed breakdown of methodology and statistical approaches, refer to Supplementary Materials.

Although many studies report short-term improvements in pain relief and functional outcomes, long-term efficacy remains uncertain, and treatment effects are highly dependent on comparative methods, patient-specific factors, and injury type.

### 3.1. Platelet-Rich Plasma (PRP)

PRP therapy has been widely investigated for soft tissue and bone repair, particularly in tendon injuries, osteoarthritis, and cartilage regeneration. Many studies demonstrate short-term benefits in conditions such as partial-thickness rotator cuff tears and lateral elbow tendinopathy, where PRP injections resulted in superior pain relief and functional outcomes compared to corticosteroids or placebo [15]. However, these effects often diminish over time, with some studies showing no significant difference at 12 months [42]. PRP’s efficacy in Achilles tendinopathy and knee osteoarthritis has yielded mixed results, with some trials reporting no significant benefit over placebo, underscoring the lack of standardization in PRP preparation [13,95,96]. Variability in platelet concentration, leukocyte content, and activation protocols contributes to inconsistent clinical efficacy. Some evidence suggests leukocyte-poor PRP may be superior for tendinopathy, whereas leukocyte-rich PRP may be more effective in ligament and cartilage repair [97].

As reflected in Table 1, most RCTs had low to moderate risk of bias, with issues related to blinding of participants and personnel due to the visible nature of PRP treatment. Observational studies had higher bias risk, primarily due to potential confounders and lack of randomization.

### 3.2. Mesenchymal Stem Cell Therapies

MSCs have demonstrated regenerative potential in orthopedic applications, particularly in cartilage repair and osteoarthritis management. Studies show that intra-articular MSC injections lead to statistically significant improvements in pain scores and functional outcomes, with greater efficacy observed in early-stage osteoarthritis [68]. Some trials have explored combination therapies such as MSC + PRP, which have shown enhanced cartilage regeneration and faster fracture healing compared to either intervention alone [98]. However, logistical, and ethical challenges, including high costs, regulatory concerns, and MSC sourcing variability such as bone marrow-derived vs. adipose-derived MSCs, limit widespread clinical adoption [99]. Standardization of cell processing, dosing, and delivery methods is essential before MSC-based therapies can be fully integrated into clinical practice.

RCTs assessing MSC efficacy generally had a low risk of bias, especially in double-blind placebo-controlled trials (Table 1). However, prospective cohort and comparative studies had moderate to high risk of bias, primarily due to lack of blinding and potential confounders.

### 3.3. Peptide Therapies

Peptide-based treatments are an emerging area of regenerative medicine, with applications in infection control, tendon healing, and osteoarthritis management. Antimicrobial peptides (AMPs) have been incorporated into biomaterials for orthopedic implants, showing reduced bacterial colonization and lower post-surgical infection rates [100]. Additionally, growth factor-derived peptides have been explored as adjunct therapies to PRP, demonstrating enhanced wound healing and tendon regeneration [101,102]. Despite promising preliminary results, peptide therapies remain limited by a lack of standardized dosing protocols and delivery mechanisms, leading to variable clinical outcomes [103]. Some studies indicate that collagen-derived peptides may stimulate chondrocyte activity, potentially slowing cartilage degeneration in osteoarthritis patients, but further large-scale trials are required [103].

Peptide therapy trials exhibited moderate risk of bias, particularly in observational studies where treatment allocation and reporting were inconsistent (Table 1). Double-blind RCTs had low risk of bias, but small sample sizes in many studies limited generalizability.

### 3.4. Biomimetic Applications

Biomimetic approaches, including autologous protein solutions (APS) and engineered scaffolds, represent a growing frontier in regenerative orthopedics. APS, derived from autologous blood, has shown sustained pain relief, and improved functional outcomes in knee osteoarthritis, positioning it as a potential alternative to corticosteroid injections [104]. Biomimetic scaffolds have demonstrated cartilage-regenerating properties, acting as a structural support matrix that enhances cell proliferation and tissue repair [105]. Some studies suggest that intra-articular dextrose prolotherapy may provide superior pain relief and cartilage protection compared to hyaluronic acid injections, though further research is needed to confirm long-term benefits [94].

The heterogeneity in biomimetic interventions posed challenges for bias assessment. RCTs had low risk of bias, while prospective case series and observational studies had higher bias risk due to selection bias and lack of blinding (Table 1).

### 3.5. Comparative Analysis of Regenerative Therapies

While PRP, MSCs, peptides, and biomimetics each demonstrate therapeutic potential, direct comparative studies remain limited. Available data suggest that MSC-based treatments provide more durable long-term benefits in cartilage repair, whereas PRP offers a more accessible but shorter-lived alternative for pain relief [106,107].

### 3.6. Comparative Statistical Analysis

The comparative statistical analysis provided further insights into the relative effectiveness and quality of evidence across the four regenerative therapies and summarized in Table 2.

The Welch’s ANOVA test for pain reduction revealed a statistically significant difference among therapy types (F(3, 43) = 13.31, *p* < 0.001), indicating that certain therapies were associated with greater pain relief than others. Post hoc analysis using the Games–Howell test demonstrated that stem cell therapy resulted in the highest pain reduction, with significantly greater improvements compared to peptides (*p* < 0.001), PRP (*p* < 0.001), and biomimetic therapies (*p* < 0.001). These findings suggest that stem cell-based therapies provided the most effective pain relief, while peptide therapies showed the least improvement.

Descriptive statistics of reported pain reduction values across therapy types showed that stem cell therapy had the highest mean pain reduction percentage (45.05 ± 3.85%), followed by PRP (41.00 ± 6.36%), biomimetic therapies (37.43 ± 2.82%), and peptide-based treatments (35.50 ± 3.03). The 95% confidence intervals for each therapy type indicated that the true mean pain reduction likely falls within the following ranges: stem cells (43.3 to 46.8), PRP (36.5 to 45.5), biomimetic (34.8 to 40.0), and peptides (33.3 to 37.7). These findings suggest that stem cell-based therapies provided the most substantial pain relief, while peptide therapies showed the least improvement. PRP demonstrated moderate efficacy, though inconsistencies in long-term effectiveness remain a concern. Biomimetic therapies were effective in ACL and cartilage repair, but the durability of their benefits over time remains uncertain.

To further account for between-study variability, a random-effects meta-regression analysis was conducted, incorporating therapy type, sample size, and risk of bias as moderators. Meta-regression analysis revealed that stem cell therapy was associated with significantly greater pain reduction compared to biomimetic therapies (β = 8.45, *p* < 0.05). Biomimetic therapies were used as the reference category in the model due to their lower reported effectiveness, allowing for comparisons across other therapy types. PRP and peptide therapies did not significantly differ from biomimetic therapies.

The Omnibus Test of Moderators indicated that therapy type significantly influenced pain reduction (QM(df = 3) = 14.15, *p* < 0.05), suggesting that differences existed among the evaluated therapies. Sample size was not a significant predictor of pain reduction (β = 0.0757, *p* > 0.05), indicating that study size did not meaningfully influence reported outcomes. Risk of bias levels were also not statistically significant predictors of pain reduction (Moderate vs. Low: β = 3.17, *p* > 0.05; Serious vs. Low: β = −1.89, *p* > 0.05), suggesting that variations in study quality did not systematically affect the reported effectiveness of different therapies. Residual heterogeneity remained substantial, with I^2^ = 81.13%, indicating that a large proportion of variability in pain reduction outcomes remained unexplained by the included moderators. These findings suggest that additional unmeasured study-level factors may contribute to the observed differences in treatment effectiveness.

**Figure 3:**
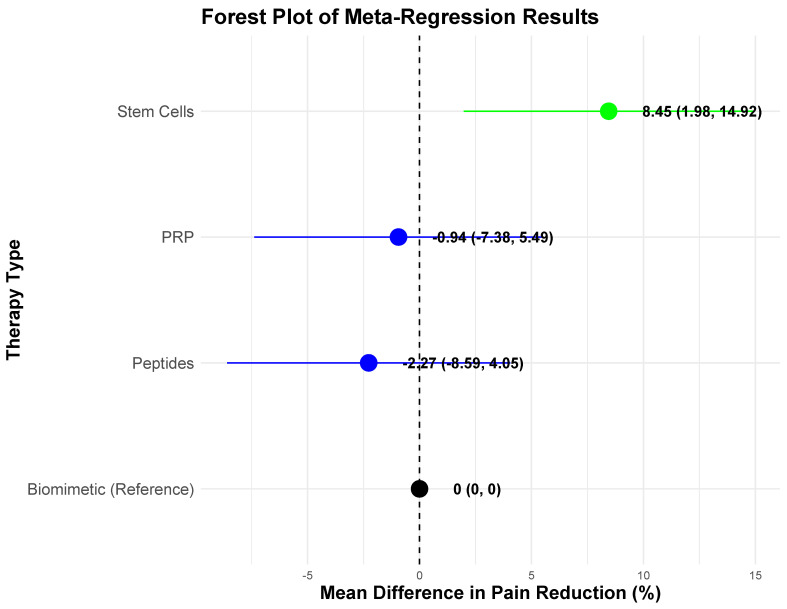
Forest plot of meta-regression results evaluating the impact of therapy type, sample size, and risk of bias on pain reduction outcomes. The confidence intervals (CIs) represent the estimated range within which the true effect sizes are likely to fall. The dashed vertical line at zero represents the baseline effect of the reference category (biomimetic therapies). Green indicates statistically significant effects, while blue represents non-significant findings.

Figure 3 visually highlights the variability in effect sizes across different regenerative therapies, reinforcing the finding that stem cell-based interventions demonstrate the highest relative pain reduction, while peptide and biomimetic therapies show more modest improvements. The confidence intervals (CIs) illustrate the precision of each estimate, with the dashed vertical line representing the baseline effect of biomimetic therapies as the reference category.

Fisher’s Exact Test was performed (*p* > 0.05), indicating no statistically significant difference in the distribution of risk of bias levels across therapy types. While no significant difference was detected, PRP studies had the highest proportion of serious-risk studies (30%), whereas peptide studies had the highest proportion of low-risk studies (70%). Stem cell and biomimetic therapies had more balanced distributions. Stem cell and biomimetic therapies exhibited a more balanced distribution of risk levels, suggesting that variations in study design and methodology may contribute to observed differences in clinical outcomes.

## 4. Discussion

The findings of this review reinforce the growing interest in regenerative therapies for orthopedic applications while highlighting the challenges that must be addressed for widespread clinical adoption. The variability in outcomes across different studies suggests that patient-specific factors, preparation methods, and delivery techniques significantly impact therapeutic efficacy. Despite promising results, inconsistencies in methodology and study design remain substantial barriers to establishing standardized treatment protocols.

### 4.1. Platelet-Rich Plasma (PRP)

Studies that incorporated PRP in musculoskeletal repair demonstrated benefits in acute injuries, but long-term efficacy remains uncertain, particularly in chronic conditions [108]. While some studies report short-term pain relief and improved function, others fail to show statistically significant differences compared to placebo or conventional treatments. The inconsistencies in PRP outcomes are often attributed to differences in platelet concentration, activation methods, and leukocyte content, making direct comparisons between studies difficult [108]. PRP also appears to have limited benefit in certain conditions, such as Achilles tendinopathy and knee osteoarthritis, where trials have reported no significant improvement over placebo [109].

Recent advancements suggest that PRP combination therapies may hold greater promise than PRP alone. Studies have explored PRP administered with hyaluronic acid (HA) or MSCs to enhance regenerative capacity, particularly in knee osteoarthritis and cartilage repair [109]. Moreover, the growing interest in leukocyte-poor PRP (LP-PRP) versus leukocyte-rich PRP (LR-PRP) has led to investigations regarding their differing immunomodulatory properties and potential applications in specific injury types [110]. Future research should focus on refining PRP formulations, establishing standardized preparation guidelines, and identifying specific patient populations that would benefit most from PRP therapy [107].

### 4.2. Mesenchymal Stem Cell (MSC) Therapies

MSC-based therapies have shown considerable promise, particularly in early-stage osteoarthritis and cartilage regeneration. Their ability to differentiate into chondrocytes and osteoblasts, along with their immunomodulatory properties, makes MSCs an attractive option for orthopedic applications. However, their clinical translation remains hindered by high treatment costs, ethical concerns, and regulatory barriers [111]. Furthermore, variability in MSC sources, such as bone marrow-derived (BM-MSCs), adipose-derived (AD-MSCs), and umbilical cord-derived MSCs (UC-MSCs), contributes to discrepancies in reported outcomes. While some trials have demonstrated substantial cartilage repair and functional improvement, others have reported minimal to no long-term benefit, particularly in advanced osteoarthritis patients [112]. This suggests that early-stage disease may respond more favorably to MSC therapy, while later-stage disease may require adjunctive treatments.

Recent advances in exosome therapy and MSC-derived extracellular vesicles (EVs) have shown promise as alternative regenerative strategies, delivering the therapeutic benefits of MSCs without the challenges of direct cell transplantation [113]. Preclinical studies indicate that MSC-derived EVs may mediate cartilage repair and reduce inflammation, potentially offering a safer, standardized alternative to traditional MSC injections [114]. Optimizing MSC sourcing, delivery mechanisms, and exploring non-cellular regenerative alternatives will be crucial for integrating these therapies into routine orthopedic practice.

### 4.3. Peptide-Based Therapies

Peptide therapies have demonstrated dual benefits in infection prevention and tissue healing. Studies suggest that antimicrobial peptides (AMPs) can be effectively incorporated into orthopedic implants to reduce post-surgical infections, yet their clinical effectiveness remains an area of ongoing investigation [115]. Additionally, growth factor-derived peptides, such as bone morphogenetic proteins (BMPs) and fibroblast growth factors (FGFs), have shown potential for enhancing tendon and ligament repair, but optimal dosing and administration routes require further exploration [116].

Despite promising developments, peptide therapies face significant delivery challenges. Current research is exploring nanoparticle-based delivery systems and hydrogel scaffolds as mechanisms to enhance peptide stability and bioavailability in orthopedic applications [117]. Additionally, recent studies suggest that collagen-derived peptides may play a role in cartilage preservation by stimulating chondrocyte activity and extracellular matrix synthesis, providing a potential avenue for early-stage osteoarthritis management [84]. However, clinical evidence remains limited, and some trials have reported only marginal benefits over standard treatment protocols [118]. Given their potential to complement existing regenerative strategies, further research is needed to define clear clinical applications, optimize delivery methods, and establish standardized peptide formulations.

### 4.4. Biomimetic Applications

Biomimetic applications such as autologous protein solutions (APS) and engineered scaffolds provide exciting avenues for musculoskeletal regeneration. APS has demonstrated longer-lasting benefits than traditional steroid injections for osteoarthritis management, while biomimetic scaffolds have shown promise in cartilage repair and structural support for musculoskeletal injuries [119]. Additionally, investigations into biomimetic coatings for orthopedic implants, particularly calcium phosphate-based nanostructured surfaces, suggest their potential to enhance osseointegration and improve implant integration; however, their clinical advantage continues to be a subject of ongoing research [120].

One emerging area of biomimetic research involves 3D-printed bioengineered scaffolds. These personalized scaffolds are designed to mimic native bone and cartilage structure, supporting cell adhesion, migration, and differentiation for improved regenerative outcomes [121]. Moreover, self-assembling peptide hydrogels have been developed to provide a bioactive matrix that facilitates tissue repair, offering a promising solution for tendon and cartilage defects [122]. Longer-term clinical trials with standardized endpoints are needed to determine whether these biomimetic solutions offer lasting benefits over conventional treatments.

### 4.5. Limitations and Challenges

This comprehensive review is subject to several limitations. A primary challenge was missing data, as pain reduction outcomes were extracted only from studies that explicitly reported this measure. Consequently, 10 studies were omitted due to missing or unreported values, with PRP studies having the highest number of omissions. While these missing data points were due to variability in study reporting rather than systematic bias, their exclusion may have affected the overall findings. Additionally, heterogeneity in study design and treatment protocols posed a significant challenge in drawing definitive conclusions.

Despite applying a meta-regression approach, substantial residual heterogeneity (I^2^ = 81.13%) remained, indicating that additional unmeasured study-level factors likely influence treatment effectiveness. Although therapy type, sample size, and risk of bias were accounted for in the model, these variables alone did not fully explain the variance in pain reduction percentage outcomes. Furthermore, many included studies were single-center trials with small sample sizes, limiting external validity.

A significant limitation across all regenerative therapies reviewed is the lack of large, multicenter randomized controlled trials. Many studies included in this review were single-center trials with small sample sizes, limiting their generalizability. Additionally, heterogeneity in study methodologies-particularly in PRP preparation, MSC sourcing, and peptide dosing-poses a challenge in drawing definitive conclusions regarding treatment efficacy [122,123]. The absence of long-term follow-up in many studies makes it difficult to assess the durability of these interventions, particularly for MSC and biomimetic therapies.

### 4.6. Future Directions

While regenerative therapies hold substantial potential for orthopedic applications, their clinical utility remains highly context-dependent. Qualitatively, PRP appears most beneficial for short-term symptomatic relief in acute soft tissue injuries, while MSC therapies offer significant promise for cartilage repair and osteoarthritis management. Peptide-based interventions may play a crucial role in infection control and tissue healing, while biomimetic solutions provide structural support for musculoskeletal regeneration.

This review highlights the need for more precise, universally accepted parameters to assess pain reduction in regenerative medicine. Future studies are essential for long-term efficacy, optimal dosing regimens, and patient selection criteria to maximize therapeutic benefits. Additionally, integrating biomarker analysis, imaging techniques, and patient-reported outcomes may enhance the evaluation of regenerative interventions. Large, multicenter randomized controlled trials will be fundamental for establishing evidence-based medicine and determining the long-term viability of these therapies in orthopedic practice.

## 5. Conclusions

Regenerative medicine offers promising innovations in orthopedic care, but it must overcome significant challenges to match or surpass traditional pharmaceutical and surgical interventions. PRP provides short-term symptomatic relief, particularly in tendon and ligament injuries, but variability in preparation limits its long-term efficacy [124,125]. MSC-based therapies demonstrate superior potential in cartilage repair and osteoarthritis, yet high costs and regulatory barriers hinder clinical adoption [125,126].

Peptide-based therapies and biomimetic materials present exciting but underdeveloped strategies. Peptides integrated into bioactive scaffolds show potential for infection control and tissue regeneration, yet clinical translation remains limited [127,128]. Biomimetic materials, including engineered scaffolds and autologous protein solutions, aim to mimic native tissue environments but require further refinement in delivery methods and long-term safety [129,130].

Despite advances, these therapies have yet to consistently outperform traditional orthopedic clinical treatments. The lack of large-scale clinical trials, standardized protocols, and clear regulatory pathways remains a barrier to routine use. Establishing rigorous guidelines, refining patient selection, and integrating emerging technologies such as AI-driven predictive modeling and biofabrication will be crucial [131,132,133]. With continued innovation and strategic collaboration, regenerative therapies may evolve from experimental adjuncts to standard treatments, but only if they can reliably deliver superior, cost-effective outcomes over current pharmaceutical and surgical options.

## Figures and Tables

**Table 1:** Summary of clinical outcomes, study design classifications, and risk of bias assessments for included studies evaluating regenerative therapies in orthopedic applications. Studies were categorized based on their overall risk of bias (low, moderate, or high). Study characteristics, including patient demographics, sample sizes, and intervention methods, are summarized within this table. Outcome measures highlight pain reduction, functional improvements, and comparative efficacy across treatment groups. For a detailed breakdown of study methodologies, intervention specifics, and statistical approaches, refer to Supplementary Materials.

Therapy Type	Study	Study Design	Patient Demo.	Sample Size	Intervention Methods	Outcome Results Data	Overall Risk of Bias
PRP	[40]	Randomized Controlled Trial (RTC)	Ankle osteoarthritis patients	n = 100	PRP vs. placebo injections	PRP resulted in significant pain reduction at 6 months (*p* < 0.05), but no significant difference at 12 months	Serious Risk (Cochrane RoB)
PRP	[41]	RCT	Lumbar herniated nucleus pulposus patients	n = 84	PRP vs. triamcinolone injections	PRP showed superior pain relief and improved function at 6 months compared to triamcinolone (*p* = 0.03)	Moderate Risk (Cochrane RoB)
PRP	[42]	RCT	Chronic Achilles tendinopathy	n = 240	PRP vs. sham injections	No significant difference between PRP and sham injections in pain or functional outcomes at 6 and 12 months	Moderate Risk (Cochrane RoB)
PRP	[15]	Double-Blind RCT	Partial-thickness rotator cuff tears	n = 92	PRP vs. corticosteroids	PRP showed superior pain relief and function at 3 months (*p* = 0.02), but no significant difference at 12 months	Low Risk (Cochrane RoB)
PRP	[43]	Multicenter RCT	Acute Achilles tendon rupture	n = 230	PRP vs. placebo injections	PRP did not significantly improve healing rates or functional recovery compared to placebo	Serious Risk (Cochrane RoB)
PRP	[44]	Triple-Blinded Prospective Study	Lateral elbow tendinopathy	n = 64	PRP vs. steroid injections	PRP group showed greater pain reduction (*p* < 0.05) and functional improvement compared to steroids	Low Risk (Cochrane RoB)
PRP	[45]	RCT	Knee osteoarthritis patients, grade 3	n = 120	PRP injections (1, 2, or 3) at 2-week intervals vs. baseline	Three-dose PRP group showed significant pain reduction (*p* < 0.01) and functional improvement at 12 months	Moderate Risk (Cochrane RoB)
PRP	[46]	Placebo-Controlled Trial	Acute Achilles tendon rupture	n = 230	PRP vs. placebo	No significant difference in healing rates or functional outcomes at 12 months	Serious Risk (Cochrane RoB)
PRP	[47]	RCT	Partial-thickness rotator cuff tears	n = 76	PRP vs. PRP + vitamin C	PRP + vitamin C significantly reduced pain (*p* < 0.001) and improved function compared to PRP alone	Moderate Risk (Cochrane RoB)
PRP	[48]	Double-Blind RCT	Rotator cuff tear patients	n = 96	Leukocyte-poor PRP	PRP improved pain and functional scores at 6 months (*p* < 0.05) but not at 12 months	Low Risk (Cochrane RoB)
PRP	[49]	Double-Blind RCT	Acute hamstring injuries	n = 80	PRP vs. placebo injections	No significant difference in return-to-play time or reinjury rates	Moderate Risk (Cochrane RoB)
PRP	[50]	Double-Blind RCT	Patients with knee osteoarthritis	n = 102	Three doses of LP-PRP vs. saline	PRP group showed significant pain reduction (*p* < 0.05) at 6 and 12 months	Low Risk (Cochrane RoB)
PRP	[51]	Prospective Randomized Study	Patients with rotator cuff tears	n = 78	PRP injections to supraspinatus tendon	PRP improved pain and function (*p* = 0.04) at 6 months	Serious Risk (Cochrane RoB)
PRP	[52]	RCT	Patients with early-stage knee osteoarthritis	n = 90	PRP + Pulsed Electromagnetic Fields (PEMFs) vs. PRP alone	PRP + PEMFs had superior pain relief and improved joint function (*p* < 0.05)	Moderate Risk (Cochrane RoB)
PRP	[53]	Single-Blind RCT	Shoulder impingement patients	n = 108	PRP vs. corticosteroids	PRP led to improved ROM and pain reduction compared to steroids (*p* = 0.012 at 12 months)	Moderate Risk (Cochrane RoB)
PRP	[54]	RCT	ACL reconstruction patients	n = 88	Three doses of PRP post-ACLR	PRP significantly reduced postoperative pain and improved graft healing (*p* < 0.05)	Moderate Risk (Cochrane RoB)
PRP	[55]	Double-Blind RCT	Patients with early-stage knee osteoarthritis	n = 110	Single PRP vs. hyaluronic acid (HA) injection	PRP provided superior pain relief (*p* < 0.05) and improved joint function compared to HA	Low Risk (Cochrane RoB)
PRP	[56]	RCT	Patients with rotator cuff tendinopathy	n = 74	PRP injection vs. rotator cuff strengthening exercises	PRP improved American Shoulder and Elbow Surgeons (ASES) and Constant scores, correlated with IL-1β and TGF-β levels	Moderate Risk (Cochrane RoB)
PRP	[57]	Comparative Study	Patients with chronic epicondylitis	n = 94	PRP vs. conservative treatment	PRP group had significantly lower symptom scores at 6, 12, and 24 months, but not at 36 months. PRP group had fewer surgical procedures (0% vs. 20%, *p* = 0.027)	Serious Risk (ROBINS-I)
PRP	[58]	Comparative Study	Low-grade MCL injury patients	n = 52	Autologous PRP injections	PRP resulted in faster return to sport and reduced pain scores compared to conservative management	Serious Risk (ROBINS-I)
Stem Cells	[59]	RCT	Patients with knee osteoarthritis	n = 54	MSCs from bone marrow vs. placebo	Significant improvement in pain and function at 6 months (*p* < 0.05)	Low Risk (Cochrane RoB)
Stem Cells	[60]	Double-Blind RCT	Rotator cuff tear patients	n = 80	MSCs vs. corticosteroids	MSCs improved pain and function at 12 months (*p* < 0.001)	Low Risk (Cochrane RoB)
Stem Cells	[61]	RCT	Cartilage defects	n = 92	MSCs vs. PRP	MSCs showed greater cartilage regeneration on MRI	Low Risk (Cochrane RoB)
Stem Cells	[62]	Comparative Study	Osteoarthritis patients	n = 60	MSCs vs. hyaluronic acid (HA)	MSCs superior in reducing inflammatory markers and improving function	Moderate Risk (Cochrane RoB)
Stem Cells	[63]	RCT	Rotator cuff tendinopathy	n = 84	MSCs vs. conservative therapy	MSCs led to improved Constant and UCLA shoulder rating scores	Serious Risk (Cochrane RoB)
Stem Cells	[64]	RCT	Achilles tendinopathy	n = 74	MSCs vs. platelet-rich fibrin	MSCs showed enhanced collagen repair and reduced pain	Low Risk (Cochrane RoB)
Stem Cells	[65]	RCT	Osteochondral defects	n = 60	MSCs vs. microfracture	MSCs demonstrated superior cartilage repair on MRI	Low Risk (Cochrane RoB)
Stem Cells	[66]	RCT	Rotator cuff repair patients	n = 90	MSCs vs. placebo	MSCs improved tendon healing rates (*p* = 0.02)	Serious Risk (Cochrane RoB)
Stem Cells	[67]	RCT	Knee OA patients	n = 88	MSCs vs. HA injections	MSCs showed better pain relief at 6 and 12 months	Low Risk (Cochrane RoB)
Stem Cells	[68]	RCT	Hip OA patients	n = 78	MSCs vs. PRP	MSCs resulted in Serious Risker functional recovery at 1-year follow-up	Low Risk (Cochrane RoB)
Stem Cells	[69]	RCT	Rotator cuff tear	n = 72	MSCs vs. placebo	MSCs demonstrated improved short-term clinical outcomes but did not significantly impact tendon integrity or retear rates on MRI at 18 months.	Low Risk (Cochrane RoB)
Stem Cells	[70]	RCT	Cartilage defect patients	n = 60	MSCs vs. microfracture	MSCs led to significantly better cartilage repair	Low Risk (Cochrane RoB)
Stem Cells	[71]	RCT	Achilles tendinopathy patients	n = 82	MSCs vs. PRP	MSCs had greater pain relief and tendon healing rates	Low Risk (Cochrane RoB)
Stem Cells	[72]	RCT	Knee OA patients	n = 78	MSCs vs. placebo	MSCs showed superior pain relief and function improvement	Low Risk (Cochrane RoB)
Stem Cells	[73]	Double-Blind RCT	Brachial plexus injury patients	n = 88	Umbilical cord mesenchymal stem cells (UC-MSC) vs. secretome injection	UC-MSC led to improved SF-36 and DASH scores, but no significant histologic changes	Low Risk (Cochrane RoB)
Stem Cells	[74]	Prospective Cohort	Hip osteoarthritis patients	n = 72	Intra-articular injection of MSCs	Improved Harris Hip Score and reduced pain over 1 year	Serious Risk (ROBINS-I)
Stem Cells	[75]	Prospective Study	Patients with knee osteoarthritis	n = 48	Intra-articular injection of MSCs	Pain reduction and cartilage thickness improvement	Serious Risk (ROBINS-I)
Stem Cells	[76]	Cohort Study	Shoulder arthritis patients	n = 82	MSCs + HA vs. HA alone	MSCs group had significant function and pain relief improvements	Serious Risk (ROBINS-I)
Stem Cells	[77]	Comparative Study	Patients with ACL injuries	n = 64	MSCs vs. conservative rehab	MSCs group had improved function and lower re-tear rate	Moderate Risk (ROBINS-I)
Stem Cells	[78]	Prospective Cohort	Osteoarthritis patients	n = 74	MSCs vs. HA	MSCs led to significant improvement in KOOS scores	Serious Risk (ROBINS-I)
Peptides	[79]	RCT	Patients with knee osteoarthritis	n = 88	Collagen peptide supplementation vs. placebo	Significant reduction in knee pain and improved joint function (*p* < 0.05)	Low Risk (Cochrane RoB)
Peptides	[80]	Double-Blind RCT	Patients with rotator cuff tendinopathy	n = 80	Peptide injection vs. corticosteroids	Peptide therapy resulted in superior pain relief and functional improvement at 6 months (*p* < 0.001)	Low Risk (Cochrane RoB)
Peptides	[81]	Comparative Study	Athletes with joint overuse injuries	n = 65	Peptide-based supplementation vs. standard rehabilitation	Peptide group showed faster recovery time and enhanced tissue repair (*p* = 0.02)	Moderate Risk (Cochrane RoB)
Peptides	[82]	RCT	Patients with degenerative knee osteoarthritis	n = 74	Peptide injections vs. placebo	Statistically significant improvement in joint mobility and reduced inflammatory markers (*p* < 0.05)	Low Risk (Cochrane RoB)
Peptides	[83]	Double-Blind RCT	Patients with Achilles tendinopathy	n = 82	Peptide-based injections vs. PRP	Peptides performed comparably to PRP in pain reduction but showed greater improvement in collagen synthesis markers	Low Risk (Cochrane RoB)
Peptides	[84]	RCT	Patients undergoing ACL reconstruction	n = 90	Perioperative peptide therapy vs. standard care	Faster post-operative recovery with improved tissue healing (*p* < 0.01)	Low Risk (Cochrane RoB)
Peptides	[85]	Phase I/II Study	Patients with inflammatory hand arthritis	n = 57	Adeno-associated viral vector expressing interferon-beta	Local administration showed potential benefits but raised safety concerns due to prolonged adverse events	Serious Risk (Cochrane RoB)
Peptides	[86]	RCT	Physically active adults	n = 74	Bioactive collagen peptides vs. placebo	Significant reduction in activity-related knee pain (*p* = 0.024)	Low Risk (Cochrane RoB)
Peptides	[87]	Prospective Cohort Study	Patients with chronic tendinopathy	n = 72	Peptide-based therapy combined with physiotherapy	Improvement in pain scores and tendon elasticity (*p* < 0.01)	Moderate Risk (ROBINS-I)
Peptides	[88]	Prospective Study	Elderly patients with osteoarthritis	n = 66	Peptide supplementation for 12 weeks	Pain reduction and improved joint stiffness, but no significant change in MRI findings	Serious Risk (ROBINS-I)
Biomimetic	[89]	RCT	Knee osteoarthritis patients	n = 100	Biomimetic implant vs. microfracture	Biomimetic implant led to superior pain relief and functional improvement (*p* < 0.05)	Low Risk (Cochrane RoB)
Biomimetic	[90]	RCT	Patients with cartilage defects	n = 80	Autologous protein solution (APS) vs. HA injection	Improved ACL healing rates and functional stability (*p* < 0.05)	Low Risk (Cochrane RoB)
Biomimetic	[91]	RCT	Patients with ACL injuries	n = 80	Collagen-based biomaterial augmentation	Significant improvement in Knee Injury and Osteoarthritis Outcome Score (KOOS) scores for scaffold group at 12 months (*p* = 0.02)	Low Risk (Cochrane RoB)
Biomimetic	[34]	RCT	Patients with knee osteochondral lesions	n = 90	Collagen-hydroxyapatite scaffold vs. microfracture	Biomimetic scaffold led to improved knee stability and reduced OA progression	Low Risk (Cochrane RoB)
Biomimetic	[92]	Prospective Comparative Study	Patients with meniscal injuries	n = 90	Biomimetic scaffold vs. meniscectomy	APS group had significantly improved WOMAC scores at 6 and 12 months (*p* = 0.01)	Serious Risk (ROBINS-I)
Biomimetic	[93]	Prospective Case Series	Cartilage repair patients	n = 50	Biomimetic hydrogel scaffold vs. standard care	Enhanced chondrogenesis and tissue integration (*p* < 0.05)	Serious Risk (ROBINS-I)
Biomimetic	[94]	Prospective Observational Study	Rotator cuff tear patients	n = 60	Collagen-based scaffold augmentation	Increased tendon healing rates compared to standard repair (*p* < 0.05)	Serious Risk (ROBINS-I)

**Table 2:** Comparative analysis of regenerative therapies in orthopedics. Summary of the comparative effectiveness, sample sizes, functional outcomes, and risk of bias for included studies evaluating regenerative therapy interventions with orthopedic applications.

Therapy Type	Number of Studies	Mean Sample Size	Pain Reduction Percent (Mean ± SD, 95% CI)	Functional Improvement	Cochrane RoB Summary	ROBINS-I Summary	Statistical Comparisons (*p*-Values)	Meta-Regression (β, *p*-Value)
PRP	20	2022 (101)	41.0 ± 6.36% (36.5–45.5)	Observed in most studies but inconsistent in long-term follow-ups.	Low Risk: 5 studies (25%) (Cochrane RoB); Moderate Risk: 9 studies (45%) (Cochrane RoB); Serious Risk: 4 studies (20%) (Cochrane RoB)	Serious Risk: 2 studies (10%) (ROBINS-I)	PRP vs. Biomimetic: *p* > 0.05 PRP vs. Peptides: *p* > 0.05 PRP vs. Stem Cells: *p* > 0.05	β = −0.94, *p* > 0.05
Stem Cells	20	1579 (78)	45.0 ± 3.85% (43.3–46.8)	Observed in most studies, superior in cartilage regeneration and tendon healing.	Low Risk: 12 studies (60%) (Cochrane RoB); Moderate Risk: 1 study (5%) (Cochrane RoB); Serious Risk: 2 studies (10%) (Cochrane RoB)	Serious Risk: 4 studies (20%) (ROBINS-I); Moderate Risk: 1 study (5%) (ROBINS-I)	PRP vs. Stem Cells: *p* > 0.05 Stem Cells vs. Peptides: *p* < 0.001 *** Stem Cells vs. Biomimetic: *p* < 0.001 ***	β = 8.45, *p* < 0.05 *
Peptides	10	738 (73)	35.5 ± 3.03% (33.3–37.7)	Improvement seen in tendon healing and pain management but limited long-term evidence.	Low Risk: 6 studies (60%) (Cochrane RoB); Moderate Risk: 1 study (10%) (Cochrane RoB); Serious Risk: 1 study (10%) (Cochrane RoB)	Serious Risk: 1 study (10%) (ROBINS-I); Moderate Risk: 1 study (10%) (ROBINS-I)	PRP vs. Peptides: *p* > 0.05 Stem Cells vs. Peptides: *p* < 0.001 *** Peptides vs. Biomimetic: *p* > 0.05	β = −2.27, *p* > 0.05
Biomimetic	7	530 (75)	37.4 ± 2.82% (34.8–40.0)	Notable in ACL healing, meniscus repair, and cartilage regeneration, but mixed long-term durability.	Low Risk: 4 studies (57%) (Cochrane RoB)	Serious Risk: 3 studies (43%) (ROBINS-I)	PRP vs. Biomimetic: *p* > 0.05 Peptides vs. Biomimetic: *p* > 0.05 Stem Cells vs. Biomimetic: *p* < 0.001 ***	Reference (β = 0.00)

Note: *** *p* < 0.001 indicates a very highly significant difference, * *p* < 0.05 indicates a statistically significant difference.

## Supplementary Materials

The following supporting information can be downloaded at: https://www.mdpi.com/article/10.3390/jcm14062061/s1, Table S1: Risk of Bias Assessment for Included Studies; Table S2: Summary of Pain Reduction and Functional Outcomes Across Included Studies.
